# A Byzantine Clinical History

**Published:** 1895-04-27

**Authors:** 


### A Byzantine Clinical History.

In the 117th section of his "Organon," Samuel Hahnemann notices the curious idiosyncrasies which cause otherwise healthy persons to be strangely affected by agents which make no such impression on the majority. Some men, for example, faint at the smell of roses. In such cases, he observes, the agent tends to produce the same result in everyone, and, therefore, tends also, according to the homœopathic law, to cure similar affections in everyone. Roses, for instance, make very few people faint, but this is enough to show that they are the homœopathic remedy for fainting in all cases. He supports this theory, which seems to include the crucial instance of the action of quinine in ague, by a quotation from the Byzantine historians: "Thus the Princess Maria cured her brother, the Emperor Alexius, who suffered from faintings, by sprinkling him with rose-water in the presence of his aunt Eudoxia."

Hahnemann's quotation is not quite correct, but, apart from its bearing upon homœopathy, the case alluded to may be not without interest to our readers. The patient was one of the ablest of the Byzantine Emperors, and his illness is described by his daughter Anna, who, as she is careful to inform us, was fully acquainted with all the medical knowledge of the period.

Alexius, during the latter part of his life, was a martyr to gout. The "procatarctic" or exciting causes of the disease were, according to Anna, three in number. First, a severe bruise of the left knee, which he received while playing at ball on horseback. He neglected rest, and the pain of the injury drew the morbid humours down into the limb. A second cause was the fatigue and mental anxiety he underwent while the Crusaders were encamped at Constantinople. For this, says the Princess, the French nobles were to blame. "The French are by nature shameless, and without self-restraint, while in loquacity they surpass the rest of mankind." They used to come one after the other to talk to the Emperor, who, determined to give them no excuse for a quarrel, would remain standing and conversing with each from evening till midnight, or later, while his courtiers had to lean against the walls or furniture to keep from falling. The third cause was not only "procatarctic" but "synectic" (*i.e.*, in continuous action), but she declines to say what it was. She probably refers to the domestic intrigues, in which she herself took a large part.

His final illness began on a windy day at the beginning of A.D. 1118. He had attended the races in the Hippodrome, and the cold air drove the morbid humours from his extremities into one of his shoulders. A consultation was held, at which the Princess Anna herself presided. Most thought it was nothing serious, "only Nicholas Callicles expressed his fear that the humours, having left the extremities, might, if not removed by evacuants, fly to one of the principal organs, even the heart, and cause an incurable lesion. But we believed him not, for we did not wish to believe him." Nicholas advised the administration of a purge, but the Emperor had never been wont to take purgatives, or indeed any form of medicine. "I (says Anna) voted with Callicles, but the majority were against us." The patient was therefore left alone and seemed to recover. "But six months later I heard him complain to the Empress, 'Whatever is the matter with my breathing? I want to take a deep breath to throw off the anxious feeling about my heart, but can never manage it, for there seems a load like a heavy stone pressing there which stops me when I begin. And to tell you something more, O dearest sharer of my cares and counsels, I often feel giddy, and have a catch in my breath.' When the Empress heard this she was seized, as it were, with the same disease." Another consultation was held, and the Emperor's pulse was found to be irregular and unequal, which the physicians attributed to an over-heating of the heart caused by the anxieties of government. The shortness of breath increased, especially on lying down; he could not lie at all on one side, and his sleep was interrupted by spasms of suffocation. At a third consultation Callicles again advised a purgative, but was again outvoted, so the Emperor was bled instead, and received a mixture containing pepper. This was followed by slight temporary improvement, but two days later he was worse than ever. He could not lie down, sleep, or take nourishment. "My mother sat continually at the bed's head and held him up. Her tears were more copious than the waters of the Nile." Dropsy set in, with swelling of the legs, which the physicians treated by applying a cautery. His throat, tongue, and palate became swollen, so that he could hardly swallow. "God knows the trouble I took with his food, pounding it up, and softening it with my own hands." On the eleventh day diarrhœa was added to his other evils. Finally, on August 15th, in the week in which the Church celebrates the falling asleep of the immaculate Mother of God, the physicians, seeing that the end was near, took their departure, and the Empress Irene and three daughters, Anna, Mary, and Eudoxia, were left alone with the patient. The Empress was exhausted with weeping. The Princess Mary was trying to give him water from a vessel with a spout, for he could not use a cup owing to his swollen tongue, &c. "I was diligently feeling his pulse, turning from time to time to comfort my mother." The Emperor had repeated fainting fits, from the second of which he recovered "on being sprinkled with cold water and rose water by my dearest sister Mary." The Empress was in such a state that she seemed likely to die with her husband. "My dearest sister Mary stood between my mother and the Emperor so as to prevent her seeing him. I carefully observed the state of his pulse, about which my mother often inquired. When I felt its force diminish, and the motion of the artery cease entirely, I bent my head as if fainting, looked on the ground without a word, and covering my face with my hands, turned aside and wept." We may omit the pathetic account of the grief of the family, which, like the rest of the picture, is perhaps somewhat over-painted, for, according to rival historians, Anna and her mother embittered the Emperor's last hours by trying to make him disinherit his son in favour of his son-in-law. With regard to Hahnemann's quotation, we may notice that he has not only mistaken the relationship, which is perhaps excusable, but also omits to mention that the princess used, as well as rose water, a commoner fluid, which has been a recognised remedy for fainting from the earliest ages.

